# Modern Micro and Nanoparticle-Based Imaging Techniques

**DOI:** 10.3390/s121114792

**Published:** 2012-11-02

**Authors:** Marketa Ryvolova, Jana Chomoucka, Jana Drbohlavova, Pavel Kopel, Petr Babula, David Hynek, Vojtech Adam, Tomas Eckschlager, Jaromir Hubalek, Marie Stiborova, Jozef Kaiser, Rene Kizek

**Affiliations:** 1 Central European Institute of Technology, Brno University of Technology, Technicka 3058/10, CZ-616 00 Brno, Czech Republic; E-Mails: marketa.ryvolova@seznam.cz (M.R.); chomoucka@feec.vutbr.cz (J.C.); drbohla@feec.vutbr.cz (J.D.); paulko@centrum.cz (P.K.); petr_babula@email.cz (P.B.); d.hynek@email.cz (D.H.); vojtech.adam@mendelu.cz (V.A.); hubalek@feec.vutbr.cz (J.H.); kaiser@fme.vutbr.cz (J.K.); 2 Department of Chemistry and Biochemistry, Faculty of Agronomy, Mendel University in Brno, Zemedelska 1, CZ-613 00 Brno, Czech Republic; 3 Department of Paediatric Haematology and Oncology, 2nd Faculty of Medicine Charles University in Prague and University Hospital Motol, Prague, Czech Republic; E-Mail: tomas.eckschlager@fnmotol.cz; 4 Department of Biochemistry, Faculty of Science, Charles University, Albertov 2030, CZ 128 40 Prague 2, Czech Republic; E-Mail: stiborov@yahoo.com

**Keywords:** imaging techniques, nanoparticles, fluorescence, magnetic resonance imaging

## Abstract

The requirements for early diagnostics as well as effective treatment of insidious diseases such as cancer constantly increase the pressure on development of efficient and reliable methods for targeted drug/gene delivery as well as imaging of the treatment success/failure. One of the most recent approaches covering both the drug delivery as well as the imaging aspects is benefitting from the unique properties of nanomaterials. Therefore a new field called nanomedicine is attracting continuously growing attention. Nanoparticles, including fluorescent semiconductor nanocrystals (quantum dots) and magnetic nanoparticles, have proven their excellent properties for *in vivo* imaging techniques in a number of modalities such as magnetic resonance and fluorescence imaging, respectively. In this article, we review the main properties and applications of nanoparticles in various *in vitro* imaging techniques, including microscopy and/or laser breakdown spectroscopy and *in vivo* methods such as magnetic resonance imaging and/or fluorescence-based imaging. Moreover the advantages of the drug delivery performed by nanocarriers such as iron oxides, gold, biodegradable polymers, dendrimers, lipid based carriers such as liposomes or micelles are also highlighted.

## 1. Introduction

**Figure 1.**
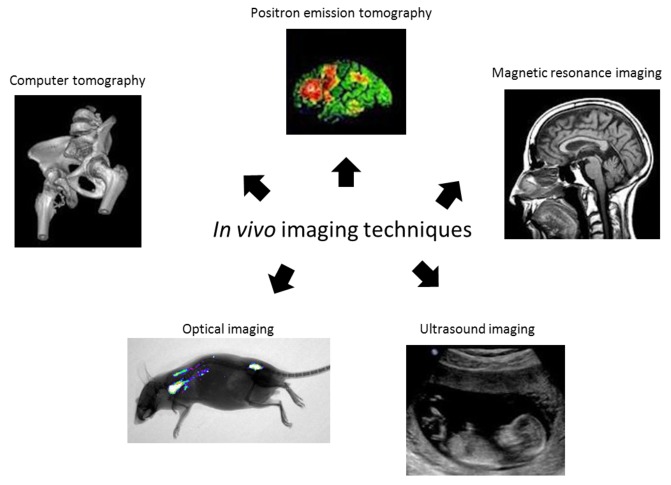
Main *in vivo* imaging techniques.

Imaging techniques play an important role in the medical care of all organ systems and better and increased research in medical imaging may be beneficial for the complete health and disease management process. Medical imaging covers many different imaging modalities: X-ray-based methods such as radiography and Computed Tomography (CT), Magnetic Resonance Imaging (MRI), Ultrasound (US), Nuclear medicine with Positron Emission Tomography (PET) and Single Photon Emission Computed Tomography (SPECT), and several optical imaging methods (Figure 1).

**Figure 2.**
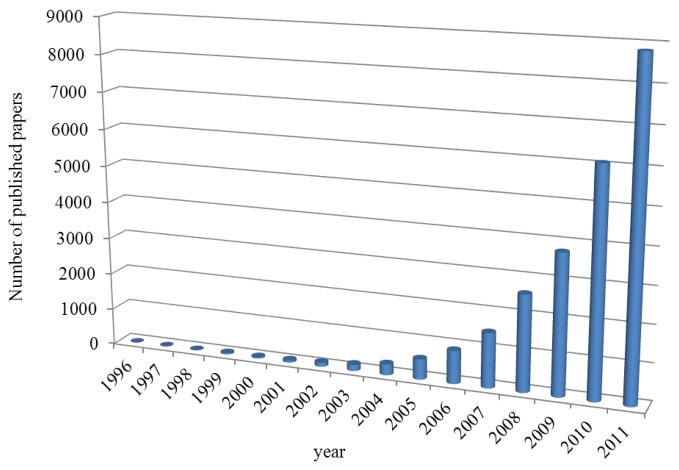
Published items each year containing “imaging and nanoparticle*” in title.

The important and exciting progress in biotechnology, nanomedicine and new innovative therapies is in many cases highly dependent on the integration of the medical imaging into standard clinical practice. The development of new materials of nanometer dimensions for biomedical applications has been in the focus in the last few years. Their applications became important in medicine, targeted therapies and diagnostics. Modern materials like nanowires [1], quantum dots [2] carbon nanotubes [3], nanoparticles [4,5] or nanomaterials [6] (Figure 2) are in the centre of attention due to the fact that the mechanical, chemical, electrical, optical, magnetic, electro-optical and magneto-optical properties of these particles are different from their bulk properties and depend on the particle size. Nanoparticles (NPs) have been developed as an important approach for delivery of conventional drugs, recombinant proteins, vaccines and more recently, nucleotides. NPs and other colloidal drug-delivery systems modify the kinetics, body distribution and drug release of an associated drug.

## 2. Magnetic Resonance Imaging (MRI)

### 2.1. Magnetic Resonance Imaging by Nanoparticles

Magnetic nanoparticles (MNPs) for medical and biological applications are being of great interest due to their unique properties. MNPs can be divided into paramagnetic, ferromagnetic and superparamagnetic particles. Superpara- and ferromagnetic particles are composed of a magnetic core and a surface coating. Paramagnetic particles are mainly based on chelates of paramagnetic ions with no explicit core and surface coating. Thus, their influence on magnetic resonance imaging (MRI) contrast is rather different from that of superpara- and ferromagnetic particles [7,8].

The switchable magnetic properties of superparamagnetic nanoparticles (SPIONs) make these materials useful for magnetic drug targeting [9], cell tracking [10], hyperthermia [11] and medical imaging [12,13]. In addition, MNPs are efficiently used for DNA, protein or cell labeling and their subsequent purification in a magnetic field [14]. MRI on its own already offers outstanding soft tissue contrast. The ongoing development of MNPs as contrast agents (CAs) further enhances image contrast. New magnetic contrast agents can be size-tailored to accumulate in specific organs or their surface can be specifically functionalized to target cells (tumor, transplanted cells). Hence, the contrast of MRI is significantly enhanced and diseases can be potentially detected at an earlier stage. Most commonly, a paramagnetic CA, usually a gadolinium-based compound, is used [15]. Gadolinium-doped tissues and fluids appear extremely bright in MR images, and for this reason paramagnetic CAs are called positive CAs.

### 2.2. Biological Application of Superparamagnetic Particles

More recently, superparamagnetic CAs based on iron oxide MNPs have become commercially available. The regions where such agents are delivered appear darker, and therefore, they are called negative CAs. The big advantage of this type of CAs is their higher sensitivity that is expected to reach single cell level [16]. Iron oxides with core/shell structures are the most widely used as sources of magnetic materials [17]. Iron oxides have several crystalline polymorphs known as α-Fe_2_O_3_ (hematite), β-Fe_2_O_3_, γ-Fe_2_O_3_ (maghemite), ε-Fe_2_O_3_, Fe_3_O_4_ (magnetite) and some others (amorphous and high pressure forms) [18]. Nevertheless, only maghemite and magnetite have found the greatest interest of bioapplications [19]. Readily, carbonyl iron, which is well-known material with a unique form of elemental iron because of its small particle size, was also used as magnetic core [20].

The surface of MNPs may be coated with number of different functionalities, depending upon the coating material and the reactive groups presented on the targeting ligand. It is desirable that MNPs retain sufficient hydrophilicity and, with coating not exceed 100 nm in size, to avoid rapid clearance by the reticuloendothelial system (RES) [21]. It was found that the surface functionalization also plays a key role in nanoparticle toxicity [17]. Jain *et al.* investigated MNPs consisting of an iron-oxide magnetic core coated with oleic acid and stabilized with a Pluronic^®^ or Tetronic^®^ block copolymer as an effective cancer theranostic (therapy and diagnostic) agent, *i.e.*, an agent with combined drug delivery and imaging properties [22]. The authors revealed that these block copolymers, mainly their molecular structures and weight, significantly influence the physical properties of MNPs, their interactions with biological environment following intravenous administration, and ability to localize to tumors. Schweiger *et al.* investigated γ-Fe_2_O_3_ MNPs for application as CAs for MRI and as switchable drug delivery vehicles [23]. Namely, the authors studied colloidal stability and cytotoxic effects of these MNPs in adenocarcinomic epithelial cells. They prepared poly (ethylene imine)-g-poly (ethylene glycol) (PEGPEI) as stabilizer for iron oxide MNPs in comparison to branched PEI. Amiri *et al.* presented in their work a series of maghemite/polymer composite ferrofluids with variable magnetic core size, which showed a good efficiency as MRI CA [16]. These ferrofluids were biocompatible and can be proposed as possible platforms for multifunctional biomedical applications. They contain anchoring groups for biofunctionalization, can incorporate fluorescent dyes, and have shown low cellular toxicity. Kumagai *et al.* developed block copolymer-coated magnetite nanoparticles for pancreatic cancer imaging, by means of a chelation between the carboxylic acid groups in PEG-poly (aspartic acid) block copolymer and Fe on the surface of the iron oxide nanoparticles [24].

Masoudi *et al.* synthesized *ex situ* PEG coated iron oxide NPs to be used as potential MRI contrast agents for early detection of lymph nodes and their metastases [25]. According to the important role of nanoparticle size in biodistribution, magnetite nanoparticles coated with different PEG concentrations up to 10/1 PEG/iron oxide weight ratio in an *ex situ* manner incomplete sentence—has no conclusion. To predict the PEG-coated nanoparticle behavior in biological media, such as the blood stream or tissues, colloidal stability was evaluated to estimate the coating endurance under different conditions. Martelli and coworkers synthesized a simple and stable nanoprobe exhibiting magnetic and fluorescent properties for detection of lymph node homing of tumor-specific dendritic cells in a murine breast cancer model [26]. The mechanism of synthesis relies on the covalent coupling of modified organic fluorophores with chitosan, which strongly interact with the surface of the ferric oxide NPs. Jiang *et al.* investigated targeted MR CA transferrin-conjugated SPIONs (Tf-SPIONs) for brain glioma detection [27]. They observed the obvious contrast change of brain glioma before and after administration of Tf-SPIONs in C6 glioma rat model *in vivo* on T2 weighted imaging.

Among the wide variety of iron oxide nanoparticles which are routinely used as MRI CAs, magnetoliposomes (MLs) may be included. The flexibility of the lipid coating allows an efficient functionalization, enabling bimodal imaging or the use of MLs as theranostic agents. Especially the large MLs containing several magnetite cores encapsulated within a single large liposome were found to be highly efficient. A special type of MLs, biogenic MLs, can also be efficiently used in this regard, with potential applications in cancer treatment and imaging. In small MLs the lipid bilayer is immediately attached onto a solid magnetite core. The flexibility of the lipid bilayer allows the incorporation of poly(ethylene glycol)–lipid conjugates to increase blood circulation times and be used as bone marrow contrast agents. Cationic lipids can also be incorporated, leading to high cell uptake and associated strong contrast generation in MRI of implanted cells [28].

## 3. Fluorescence-Based Imaging Techniques

In the group of fluorescence-based techniques fluorescence microscopy, flow cytometry, cell sorting fluorescence correlation spectroscopy as well as particle tracking velocimetry may be included. Historically, biological research is based on optical imaging, in particular microscopy, but also several chromophoric assays and photographic approaches. With the recent emergence of methods appropriate for bio-marker *in vivo* staining, such as bioluminescence, fluorescent molecular probes and proteins, as well as nanoparticle-based targeted agents, significant attention has been shifted toward *in vivo* interrogations of different dynamic biological processes at the molecular level. This progress has been largely supported by the development of advanced tomographic imaging technologies suitable for obtaining volumetric visualization of bio-marker distributions in small animals at a whole-body or whole-organ scale, an imaging frontier that is not accessible by the existing tissue-sectioning microscopic techniques due to intensive light scattering beyond the depth of a few hundred microns [29].

### In Vivo Imaging by Fluorescent Nanoparticles

Semiconductor quantum dots (QDs) are of special interest as tiny light-emitting particles on the nanometer scale that belong to new class of fluorescent probes for *in vivo* biomolecular and cellular imaging [30–33]. In comparison with organic dyes and fluorescent proteins, QDs have unique optical and electronic properties. QDs have molar extinction coefficients that are 10–50 times larger than that of organic dyes, which make them much brighter in photon-limited *in vivo* conditions. The long lifetime in the order of 10–40 ns increases the probability of absorption at shorter wavelengths, and produces a broad absorption spectrum. Further, QD emission wavelengths are size tunable. For example, CdSe/ZnS QDs of approximately 2 nm in diameter produce a blue emission, whereas QDs approximately 7 nm in diameter emit red light [34,35]. The emission wavelength was extended into the near infrared region (650 nm to 950 nm), to take advantage of the improved tissue penetration depth and reduced background fluorescence at these wavelengths. A key property for *in vivo* imaging is the unusual QD Stokes shift, which can be as large as 300–400 nm, depending on the wavelength of the excitation light [32]. In conjunction with broadband absorption and narrow emission peaks of QDs, this property allows multiplexed imaging applications in which one light source is used to simultaneously excite multicolor QDs without the need for complicated instrumentation. Another important feature is the long-term photostability of QD imaging probes, which opens the possibility of investigating the dynamics of cellular processes over time, such as continuously tracking cell migration, differentiation, and metastasis. These properties have made QDs a topic of intensive interest in cancer biology, molecular imaging, and molecular profiling [36–44]. As expected, *in vitro* investigation of QDs is of a great interest [34,45–51]. Some examples of application of QDs in imaging are given hereinafter.

A specific advantage of quantum dots for *in vivo* applications is their photostability; QDs allow images to be recorded over a longer period of time than possible with the use of fluorescent dyes or proteins due to their resistance to photobleaching. Maysinger *et al.* visualized CdSe and CdTe QDs detectable at one hour-, one day-, three days-, and seven days post intracortical injection using *in vivo* imaging techniques [52]. Mice were injected subcutaneously and scanned for fluorescence, in particular in the brain, where peak fluorescence was observable at three days post injection and persisted for seven days. Sub-cellular resolution was achieved and allowed the identification of the location of the conjugates. The researchers demonstrated internalization of their QD conjugates, with varied rate and efficacy of internalization for different cell-types. Neuronal internalization is especially challenging, and *in vivo* imaging in neural cells has important relevance to quantum dot toxicity, as this approach would allow the investigation of the neuronal immune response in real time. This approach was novel not only in the *in vivo* imaging aspects but the animal model was transgenic, and was monitored for an astrocyte-responsive luciferase reporter in addition to the injection of quantum dot conjugates. The activation of the astrocyte response was observable as an increase in bioluminescence in response to foreign nanoparticles in mice.

Zimmer *et al.* have employed NIR emitting QDs due to their ability to act as a reporter at a wavelength minimally absorbed by biological species [53]. They prepared a series of InAs/ZnSe core/shell QDs. The small core size, along with variation to the shell thickness and composition offered a range of size tunable emission wavelengths, between 750 and 920 nm. The conjugation of dihydrolipoic acid to the quantum dots allowed for the observations within the interstitial fluid in rats, where the quantum dot conjugates exited the blood vessels. This visualization of the extravasations sites is important, and has potential to interrogate the delivery mechanism of QDs to tumor cells.

Tumor growth requires a supply of nutrients from the blood stream. Angiogenesis is the process by which new vasculature establishes a blood supply to a growing tumor. Receptors, such as integrins, are highly expressed in tumor cells during angiogenesis, and diagnostics targeting such receptors can lend insight into the type and extent of diseases, including cancer. The work by Cai *et al.* detailed the use of a tri-peptide QD conjugate which specifically binds to integrin [54]. The conjugation of arginine-glycine-aspartic acid to a quantum dot with emission at 705 nm yielded a diagnostic tool specific for the integrin receptor that emits in the NIR region. *In vitro* analysis was performed on human glioblastoma and human breast cancer cells, showing specificity for integrin-positive cells only. In addition, binding was inhibited by the presence of an integrin antagonist. Injections of both the conjugates and non-specific controls through the tail vein of mice also demonstrated the specificity, with a maximum fluorescence at six hours post-injection.

Smith *et al.* showed how QDs allow for the non-invasive visualization of blood vessel development over time [55]. The conjugation of biotinylated fibrinogen to QDs showed specificity towards the membrane of blood vessels during angiogenesis. The conjugates had robust biocompatibility, staying in circulation for days without noticeable toxicity. The authors employed QDs with longer emission wavelengths to avoid native autofluorescence [56].

Jaiswal *et al.* investigated slime mold behavior, a characteristic feature of single-celled organisms in response to starvation. Four distinct cultures of AX2 amoebae cells were starved for different periods of time, each labeled with a different color QDs. The cells were then mixed with a 10-fold excess of unlabeled, non-starved cells and the QD fluorescence from the labeled cells was tracked for extended observation times. Five melanoma tumor cell populations were labeled with distinct color QDs, using cationic (Lipofectamine 2000) encapsulation, injected them into the tail vein of mice, and tracked the QD emission by multi-photon fluorescence microscopy as the cells extravasated into lung tissues [57].

An alternative to two-photon fluorescence imaging, which requires high excitation powers and a pulsed laser source, deep tissue imaging could rely on NIR fluorescence using nanocrystals that emit at 650–900 nm, a region of the optical spectrum that overlaps with tissue transparency window. NIR fluorescence could allow deep tissue imaging in animals with increased depth and with reduced background [58,59]. Krishnan *et al.* reported *in vivo* NIR imaging using epidermal growth factor (EGF) conjugated NIR emitting QDs to image the over-expression of epidermal growth factor receptor (EGFR) in tumors compared to normal expression levels in the surrounding normal tissues [60]. They found that following initial incubation, both unconjugated and EGF-coupled QDs exhibited comparable nonspecific uptake into tumor. However, the concentration of free QDs in the tumors gradually decreased, while EGF-QDs progressively accumulated in tumors up to 6 h after injection.

Recently, Kim *et al.* explored the targeting and imaging of lymphatic vessels using QD-conjugated to hyaluronic acid (QD-HA conjugates). They prepared conjugates using electrostatic attractions between anionic HA and amine functionalized DHLA-QDs, and utilized the specific properties of hyaluronic acid to target and visualize lymphatic vessels [61]. Hyaluronic acid (HA) is found in the extracellular matrix and contributes significantly to an array of processes, including cell proliferation, wound healing, metastasis, and prognosis of some malignant tumors. Authors injected HA-QDs into ears of nude mice, and showed that endothelial cells (in lymphatic vessels) could be clearly visualized for up to several days.

Choi *et al.* explored renal clearance of an intravenously administered CdSe-ZnS QDs in mice as a model system [62]. They used several size QDs capped with various hydrophilic small ligands via cap exchange and measured their half-life circulation before eventually being collected in the bladder or other organs (liver, spleen). More recently they followed up on those findings and reported on the correlation between the stabilizing ligands and the biodistribution and clearance of the hydrophilic QDs. They stabilized InAs-ZnS QDs (using InAs instead of a CdSe core) with DHLA and DHLA-PEG ligands with varying PEG chain-length. These results suggested that the biodistribution and clearance of QDs were dependent not only on the core size of the QDs but the state of the stabilizing ligands. Ultra short PEG with two units of ethylene glycol produced QDs with small hydrodynamic diameter (HD) but retained a hydrophobic character and these QDs were rapidly captured by the liver. Conversely, increasing PEG chain length from two to 22 units produced larger HD and uptake into the liver, kidney, pancreas, and lymph nodes, though with varying degrees of uptake. On the other hand, long PEG chains with larger than 22 units are highly hydrophilic and QDs remain in the vasculature for long periods of time. These findings showed that PEG, usually considered as a bio-inert polymer, affected the physical properties of QDs and their biological distribution [63,64]. QD-conjugates with overall small size could also be excreted by the kidney. These studies confirmed that proper surface functionalization and size control of QDs are essential to controlling targeting, biodistribution and excretion from the body. Growing interest in study on QDs can be further demonstrated by preparation and application of different composition of QDs such as CdPbS [65], CdSe, CdSe/CdS [66], CdSeS/ZnS [67], CdTe [68], CdSe [69], Ag_2_S [70,71], Ag_2_Se [72] or GdS/Eu [73].

Due to the materials used for QDs synthesis, concerns regarding their toxicity have been raised and numerous studies have been carried out [74,75]. At the same time, methods of synthesis of non- or low toxic QDs have been developed [76].

The excitation of traditional fluorescent labels such as organic dyes, fluorescent proteins and QDs usually requires the use of UV or short-wavelength radiation, which results in serious obstacles including low light-penetration depth inherent to the short-wavelength excitation light and/or low signal-to-noise ratio due to significant tissue autofluorescence. Therefore, it is highly essential to develop more efficient biolabels to overcome these limitations. One of such tags is represented by the “upconversion” paricles. In particular lanthanide doped rare-earth nanocrystals, are characterized by the conversion of long-wavelength radiation, for instance infrared or near infrared radiation, to short-wavelength radiation, usually in the visible range [77–79].

#### Multimodal Imaging

As mentioned above various imaging modalities have been exploited and are successfully employed to investigate the anatomic or functional dissemination of tissues in the body. However, no single imaging modality allows overall structural, functional, and molecular information as each imaging modality has its own unique strengths and weaknesses. The combination of two popular paging modalities that investigates the strengths of different methods might offer the prospect of improved diagnostic and therapeutic monitoring abilities.

Multimodal molecular imaging can offer a synergistic improvement of diagnostic ability over a single imaging modality. Inorganic nanoparticles such as magnetic iron oxide nanoparticles are studied as MRI contrast agents, QDs or upconversion nanoparticles serve as optical probes and/or metallic gold nanoparticles exhibit unique light scattering patterns for surface-enhanced Raman spectroscopy. Most popular nanostructured multimodal imaging probes are combinations of MRI and optical imaging modalities [76,80], however also trimodal imaging probes have also been designed by adding another imaging modality. In particular, positron emission tomography isotopes such as ^111^In or ^64^Cu, which emit gamma rays from their decay, have been most frequently used as the third probes [81].

## 4. Imaging by Microscopic Techniques

### 4.1. Light and Fluorescence Microscopy

Despite the fact that this technique is the well-known and the well-used in biology, its application in the detection of NPs is relatively limited. The first field is based on the histochemical detection of components of NPs, usually oxides of metal ions. Newer and newer procedures are being proposed and developed [82]. The second possibility is based on the immunohistochemistry, the process based on the detection of antigens in cells and tissues on the principle of monoclonal or polyclonal antibodies binding specifically to antigens [83,84]. However, this technique is usable only for functionalized NPs carrying antigenic structure. Chromogenic reporters are used for the detection of target, in our case NPs. The most common chromogenic reporters are enzymes, such as alkaline phosphatase (AP) and horseradish peroxidase (HRP). In addition, they can be detected not only by chromogenic substrates, such as 3,3′-diaminobenzidine (DAP) or 5-bromo-4-chloro-3-indolyl phosphate/nitro blue tetrazolium (BCIP/NBT), but also by fluorogenic and chemiluminiscent substrates usable in fluorescence microscopy. Light microscopy is used for the visualization of iron oxide-based NPs, which is based mostly on the processing of the semi thin section and Prussian blue/Berlin blue reaction method staining. This technique was used in the visualization of SPIONs in the human cochleae and mouse inner ear cell culture [85]. Incorporation of peroxidase into or onto NPs, which is widely used in the construction of hydrogen peroxide biosensors, may represent useful tool in the visualization of nanoparticles [86,87]. Like this modified NPs may catalyze the oxidation of peroxidase substrates to produce a color reaction at the site of NPs. Based on this NPs-peroxidase approach, Zhuang *et al.* determined the biodistribution and organ clearance of magnetic NPs in mice by visualizing and quantifying the localization of MNPs within the main organs [88]. This approach has significantly increased the sensitivity of NPs detection compared to convenient Prussian blue staining. Light microscopy may be suitably combined with next imaging techniques, such as magnetic resonance. For example, Neuwelt *et al.* compared histochemical and magnetic resonance detection of Ferumoxtran-10, a dextran-coated iron oxide nanoparticles, in patients suffering from primary metastatic malignant brain tumors [5]. Histochemistry revealed presence of Ferumoxtran-10 NPs in the reactive cells, predominantly in reactive astrocytes and macrophages, and not in tumor cells. This work demonstrates possible benefit of combination of magnetic resonance with histochemistry, which may improve our knowledge about distribution of NPs suffering from malignant disease. Compared to convenient light microscopy, fluorescence microscopy brings some advantages, especially significantly higher quality of images. Samples must be fluorescently stained despite the fact that many compounds are responsible for the intrinsic autofluorescence of sample. Autofluorescence may be used for detection of nanoparticles, as NPs are able to affect autofluorescence of cells. These effects are probably caused by the high surface area of the NPs and ability to catalyze different processes leading to the chemical modification of autofluorescent biomolecules. El-Sayed *et al.* treated two oral squamous carcinoma cell lines and one immortalized benign epithelial cell line with gold nanoparticles of different shapes, both spheres and rods and monitored changes in autofluorescence caused by soluble NADH and collagen. They significantly quenched the autofluorescence of both compounds based on the oxidation of NADH to NAD^+^ catalyzed by NPs surface and photonic absorption of light by collagen [89]. This knowledge suggests new potential of NPs in cell and tissue imaging. Fluorescence microscopy enables combination of differently labeled secondary antibodies and multifunctional cell/tissue labeling.

### 4.2. Confocal Microscopy

Conventional immunofluorescence methods may be used for imaging of NPs by confocal (and fluorescence) microscopy. Confocal microscopy is ideally suited to the investigation of biological tissue because it is able to provide high quality images from within a localized region of the cell/tissue. Immunofluorescence methods are based on the specificity of antibodies to antigen to target fluorescence probe to NPs. Nevertheless, NPs must be functionalized by biomolecules which represent a suitable antigen. Secondary, indirect, immunofluorescence is the most commonly used technique in NPs visualization and detection. On the other hand, the complexity of NPs' structure often enables binding of a fluorescent probe or more different fluorescence probes (“doped” NPs) and the NPs can subsequently be visualized directly using confocal fluorescence microscopy techniques. Luminescent nanoparticles represent one of the most interesting opportunities for confocal microscopy. Especially silica-based nanoparticles doped with fluorescence molecules [90,91], gold nanoparticles [92], and quantum dots and rods and luminescent nanocrystals called nanophosphors are discussed [93,94]. Organically modified silica (ORMOSIL) and nanoparticles doped with two-photon absorbing fluorophores, which exhibit aggregation-enhanced fluorescence, and nanophosphors (ceramic nanoparticles containing luminescent lanthanoid ions) are two most promising luminescent NPs [95–98]. The most important confocal microscopy techniques are briefly summarized in Table 1.

Studies of cell processes as well as NPs transport and localization are multiparameter problem, so it is highly necessary to examine many parameters within the cells. Due to these needs, microscope systems for multispectral imaging have been developed. Multispectral confocal microscopy enables elegant solving of these needs and enables not only monitoring the cellular uptake, transport and localization of NPs, but also connection of these processes with drug delivery systems [99].

### 4.3. Electron Microscopy

Electron microscopy is an irreplaceable tool in the determination of the size and structure of NPs. The basic application of electron microscopy methods in NPs detection/visualization including the most common scanning (SEM) and transmission electron microscopy (TEM) is summarized in Table 2.

Transmission electron microscopy is the most used technique in the characterization of NPs. TEM was used for the characterization and visualization of different types of nanostructures, such as noble metal- and metal oxide-based NPs [105–107], carbon nanomaterials [108,109], quantum dots [110–112], or polymeric nanoparticles of a dendrimer type [113]. TEM is preferentially used in the study of nanoparticle uptake, cellular compartmentation, transport and accumulation in different tissues and organs. Recently, TEM was used to investigate the presence of metal nanoparticles inside the carbon nanotubes [114]. Scanning electron microscopy is a necessary tool in the determination and structural characterization of NPs, such as size and shape, area, presence of pores, or eccentricity; in addition, SEM is highly necessary in NP quality control [115–117]. Analytical Electron Microscopy (AEM) has become an essential tool for microstructural investigations of nanostructures. It has become one of the most powerful tools to obtain structural, morphological, and compositional information at the nanometer scale by combining imaging, diffraction and spectroscopy, e.g., EDS (energy dispersive X-ray spectrometry) and Electron Energy Loss Spectrometry (EELS). Field-emission gun (FEG) electron source instruments enable highly effective study of nanostructures using various methods, such as high resolution transmission electron microscopy (HRTEM) [118,119], high-resolution Z-contrast (STEM/HAADF) imaging [120], different techniques of electron diffraction (SAED, CBED, nanodiffraction) [121], X-ray energy dispersive spectroscopy (XEDS) [122,123] and electron energy loss spectroscopy (EELS) [65,124,125].

In addition to all above-mentioned methods, there are other techniques that have found application in the imaging and visualization of NPs and nanomaterials. The most important techniques are scanning acoustic microscopy (SAM, a technique that uses focused sound to investigate, measure, or image an object and can provide data about the cytoskeleton and movement of the cells in biological and medical applications) [126,127], X-ray microscopy (XM, which uses electromagnetic radiation in the soft X-ray area and is applicable on biological samples in natural state; [128,129], scanning tunneling microscopy (STM) for imaging of surfaces at the atomic level [130–132], scanning probe microscopy (SPM, surface images under the use of physical probes) [133–136] and especially its type atomic force microscopy (AFM, an application used especially in biophysics in the monitoring of protein-ligand interactions) [133,137–139].

### 4.4. Electrochemical Microscopy

Another imaging technology, which is in the interest of scientists, is Scanning Electrochemical Microscopy (SECM). SECM can provide information about the topography and electrochemical properties of a surface. Measured samples do not have to be special prepared. Using this method it is possible to measure the cellular activity and the effects of cellular inhibitors by observing the oxygen reduction current [144], to investigate the anticancer drug sensitivity [145], to detect genetic mutations [146] or select metastatic/nonmetastatic cells base on their physiologic effects [147]. The topography and intracellular redox behavior of non-metastatic and metastatic cell lines was investigated too [148]. Liebetrau *et al.* [149] showed that SECM could record real-time morphological changes evoked by modulating the ionic strength of surrounding medium. This was done via using hydrophobic mediators (e.g., to obtain quantitative information about morphology of living cells PC12 by studying changes in the tip current arising from topography variations).

## 5. Imaging by Laser-Induced Breakdown Spectroscopy (LIBS)

Although similar to microscopy, LIBS cannot be applied to higher living organisms such as animals, but it can be potentially applied to analyze cells and therefore we believe that it should be mentioned in this review.

Laser-Induced Breakdown Spectroscopy (LIBS) is a spectrochemical analytical technique that utilizes high power densities employing focused radiation, usually from a pulsed, fixed-frequency laser, in order to generate luminous plasma from a sample (solid, liquid, and gaseous samples) [150]. During the LIBS analysis the stoichiometric sample ablation is assumed, so that the plasma composition reflects the elemental composition of the ablated target. Comparing LIBS to another, more traditional laser-ablation based techniques, *i.e.*, to LA-ICP-OES/MS highlights that LIBS setup is in general simpler and this approach is more cost reliable. Considering the capabilities for spatially-resolved analysis, LIBS gives an instantaneous signal directly related to the location at which a single ablation event occurred, while mapping using LA-ICP-OES/MS usually represents more experimental difficulties, because this technique involves sample transport, so the signal produced in the mass (or optical) spectrometer without considerable care in the analysis is not directly attributable to a specific location on the sample [151]. LIBS is uniquely capable for light-element detection, useful for material identification, depth profiling, and elemental surface mapping, and so it can solve numerous industrial and scientific problems in real-time [152–154]. On the other hand, the limits of detection (LOD) of LIBS, which in its basic configuration are typically of the order of mg·kg^−1^ or tens of mg·kg^−1^ for most of the elements, are poorer with respect to other more traditional analytical techniques (e.g., ICP-MS or ICP-OES) [146]. One of the suggested approaches to improve LIBS LODs is the double-pulse (DP) configuration. The DP-LIBS [155,156] approach was introduced by Niemax and his co-workers from ISAS Dortmund in 1991 [157], and nowadays it is a common technique used in many LIBS research laboratories. DP-LIBS allows better coupling of the laser energy to the target and to the ablated material, leading so to a more efficient production of analyte atoms in the excited state.

The necessary LIBS instrumentation can be summarized as short duration pulsed laser (or lasers in the case of DP-LIBS technique), focusing optics, collecting optics for the radiation emitted by the laser-induced plasma, a wavelength analyzer and a gated detector (capable temporarily resolved analysis), all computer controlled [152].

### LIBS in Nanoparticles Analysis

The LIBS capabilities for rapid analysis of multi-component pharmaceutical tablets were demonstrated using several examples earlier [158,159]. LIBS was evaluated as a rapid and direct method for measuring the drug content in pharmaceutical tablets. Although the tablet components are all organic, in a large fraction of cases the drug molecule contains an element which distinguishes it from the other tablet components. Measuring the drug content can therefore be carried out by quantifying this element [159].

Based on our experiences the extension of the LIBS capabilities for determination of Quantum Dots (QDs) in liquid solution is feasible. LIBS has not been used for this purpose, in spite of the fact that it could cast the light on qualitative and quantitative properties of produced nanoparticles. In our work, we prepared QDs as particles floating in liquid medium, so it has to be ensured that they are deployed equally during the time of LIBS experiment in the whole volume. Basically, there are two ways how to achieve this: circulate the medium or deposit the liquid medium on some kind of carrier medium. Circulating the medium has a disadvantage in a relatively big volume of necessary liquid (∼20 mL). On the contrary, application of liquids on suitable carrier medium needs small amount of sample (∼1 μL) and also it can be more easily manipulated. Materials suitable for carrier medium have to comply with following parameters - chemical stability, high absorption, well defined elemental composition (most important is that any spectral line of included elements does not interfere with spectral lines of elements of interests), low costs and easy manipulation.

**Figure 3.**
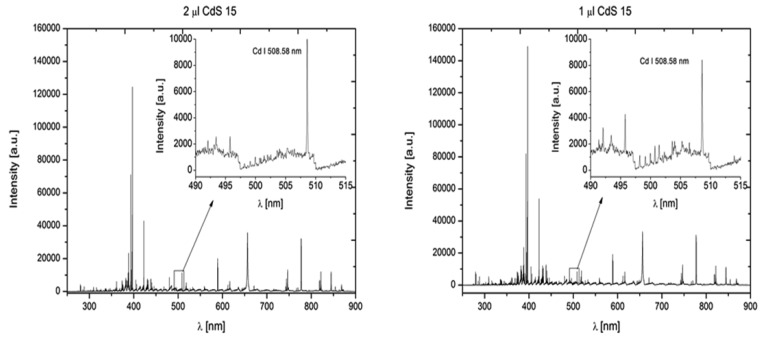
Comparison of DP-LIBS spectrum for the CdS QDs sample of 1 μL and 2 μL volume. First laser—532 nm, pulse duration ∼10 ns, perpendicular direction to the sample surface, second laser—1,064 nm, pulse duration ∼6 ns, parallel direction to the sample. Spectral lines 508.58 nm for cadmium and 276.67 nm for tellurium.

The ∼10 ns long, 532 nm laser pulse of ablation laser was directed perpendicular to the sample surface by mirrors and focused by a 30 mm focal length glass triplet. The beam of second 1,064 nm laser pulse, with a pulse width ∼6 ns was directed parallel to the sample surface to the plasma. Spectral lines 508.58 nm for cadmium and 276.67 nm for tellurium were studied (Figure 3).

## 6. *In Vivo* Imaging of Targeted Drug Delivery

Drug nanodelivery belongs to one of the most important part of nanomedicine that is already implemented. Difficulties with the drug delivery, such as low solubility and/or bioavailability, short half-life or inconvenient distribution to the target location may cause drug inefficiency. Nanoparticles designed for tumor targeted therapies consist of various components, in most cases a nanocarrier (nanovector) and a drug [160].

**Figure 4.**
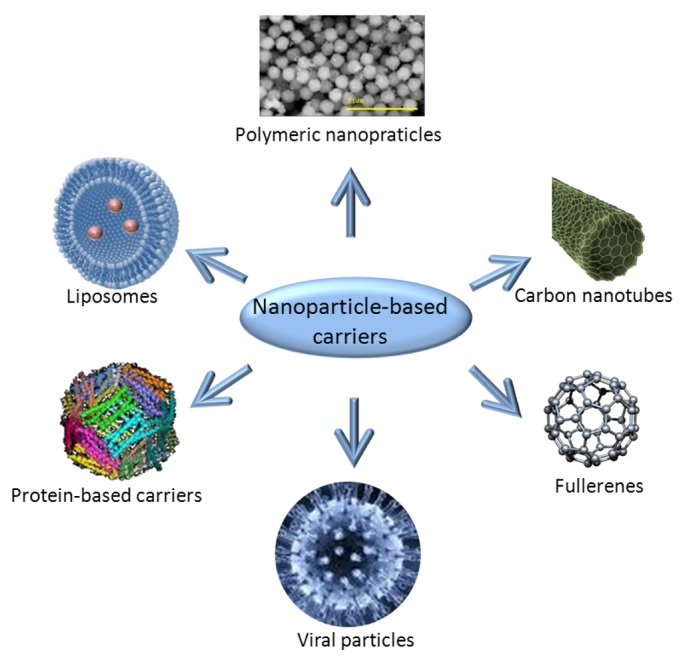
Nanoparticle-based carriers used for targeted drug/gene delivery.

Nanoparticle carriers are mostly composed of iron oxides, gold, biodegradable polymers, dendrimers, lipid based carriers such as liposomes or micelles, viruses (viral nanoparticles) or organometallic compounds [161–164]. The majority of anticancer therapeutics is water insoluble and need to be dissolved in an organic solvent that is usually toxic [165]. The low molecular weight of anticancer drugs causes their rapid excretion, requiring the administration of escalating doses [166]. The drug encapsulation in nanocarriers provides better biocompatibility. Several such engineered drugs are already used in clinical practice as registered drugs e.g., liposomal doxorubicin [167]. The main advantage of these particles in the clinic is minimum toxicity of the carrier itself and decreased toxicity of the anticancer drug [168]. Concerning the nanoparticles shapes used are nanoshells, nanorods, nanocages, nanocubes or nanotubes (Figure 4). Nanoparticle-mediated targeted delivery of drugs might significantly reduce the dosage of the drugs with keeping of the effect that reduce side effects and increase its specificity and bioavailability [12,164,169].

Therapeutic nanosystems have four unique properties: (i) they themselves may have therapeutic properties; (ii) nanosystems can be attached to targeting ligands, which yield high affinity and specificity for target cells; (iii) nanosystems can contain combination of drugs; (iv) nanosystems can bypass some of drug resistance mechanisms. By using both passive and active targeting strategies, the nanocarriers can achieve increased intracellular concentration of drugs in cancer cells while minimizing toxicity in normal cells, simultaneously enhancing anticancer effects and reducing systemic toxicity [170,171].

Targeted delivery in anticancer therapy is one of the more intensively studied areas. The above mentioned improvements are based on the enhancement of permeability and retention of the vasculature surrounding tumors caused by poor quality of newly developing tumor vessels which carry of nanoparticles to target molecules expressed by tumor cells [172]. Antibodies, peptides, nucleic acid aptamers, carbohydrates and small molecules can be used as ligands. The key features of anticancer nanoparticles are mainly their size, surface properties and in some types of them also targeting ligands. The size of nanoparticles varies between 10 to 200 nm. Nanoparticle property requirements also depend on tumor characteristics such as cancer type, clinical stage, or location [173]. The precise and fast diagnostic of therapeutic targets is one of the basic conditions for implementation of personalized medicine. For targets detection nanoparticles can be used [174,175]. Photodynamic therapy (PDT) is based on utilization of light-sensitive drugs or photosensitizers that can be preferentially localized in tumors and their therapeutic effect is activated by excitation of the photosensitizer to generate a cytotoxic species, usually singlet oxygen, free radicals, or peroxides. This caused relatively selective damage of cancer cells but spare adjacent healthy ones. There are several technical difficulties in the application of PDT, but some of them could be overcome by using of nanoparticles. Nanoparticles in PDT can be divided into passive carriers and active participants in photosensitizer excitation. Active nanoparticles are classified into photosensitizer nanoparticles, self-illuminating nanoparticles and upconverting nanoparticles [176]. QDs can transfer energy to surrounding O_2_, generate singlet oxygen with consequent toxicity to cells [177]. The quality of a photosensitising drug for PDT depends on its ability to transfer energy and/or electron to molecular oxygen and the subsequent production of reactive oxygen intermediates (ROI). Photostability, broad absorption band and large two-photon absorption cross-section of QDs are advantages for PDT. Although targeted delivery of QDs in cancer cells and tumor milieu is possible, compared with conventional photosensitising drugs, the efficiency of QDs to produce ROI induced by direct photoactivation is lower [177]. Therefore conjugates between QDs and conventional photosensitising drugs are subjects of intensive research [178].

Other application of nanoparticules is hyperthermia. Thermal ablation involves heating cells at temperatures above 55 °C, which causes coagulative necrosis and protein denaturation that kill targeted cells. The temperature of carbon nanotubes can rise to over 60 °C within 2 min when they are exposed to near infrared wavelengths of 700–1,100 nm [179]. Research has been focused on development of nanostructures that produce heat on activation by near infrared optical excitation. Such nanoparticules target specifically the tumor and selectively ablate it, leaving surrounding healthy tissue undamaged. This therapeutic modality has been shown to be efficacious for the treatment of several tumors, e.g., lung, liver, and prostate carcinoma [180]. Magnetic nanoparticles can also be used for hyperthermia. Magnetic nanoparticles can be localized in deep tissue and fixed by external magnetic field and alteration of the AC field increase the temperature of the magnetic nanoparticles [179]. Radiosensitizers are drugs that enhance the effects of radiotherapy, and some of them are metallic nanoparticles. Such nanoparticles should provide enhanced interaction cross-section with gamma photons. They increase effect of radiotherapy by scattering and/or absorbing X and γ rays causing localized damage of cancer cells and thus decreasing total radiation dose to minimize side effects [181]). This approach of using nanoparticules as radiosensitizers is termed Nanoparticle Enhanced X-ray Therapy (NEXT). It is planned to test CaF, LaF, ZnS or ZnO QD, carbon dots, liposomes with some radiosensitizers or antioxidants, nanoparticle-photosensitizer conjugates and silicon nanoparticles [182]. Radiosensitization by 1–12 nm gold nanoparticles irradiated by high energy electrons generated low energy electrons that caused increased DNA damage [183]. In another study, gold microspheres of 1.5–3.0 μm size have been tested both *in vitro* and *in vivo* studied for enhancement of X-rays that resulted in radiosensitization by gold particles [184]. Furthermore, the application of gold nanorods for therapeutic purposes has been investigated [185–187]. Contrast nanoparticules may also increase efficiency and/or safety of radiotherapy by improvement of planning by MRI [188].

Nanotechnology may also be applied in diagnostics for surgical oncology. Nanoparticles can be accumulated in solid tumors that enable one to localize tumors, detect margins, identify important adjacent structures, map sentinel lymph nodes, and detect residual tumor cells or micrometastases. Intraoperative imaging overcomes the tissue penetration problem of optical methods [189].

## 7. Outlook

Imaging techniques are progressing rapidly due to the development of new types of contrast agents as well as progress in the development of instrumentation, electronics and software. This is a multi/transdisciplinary approach combining the knowledge of chemists, biologists, physicists, and mathematicians as well as engineers and IT specialists. It is highly improbable that QDs will fully replace the well-established technologies using fluorophores or fluorescent protein-fusion, but will rather complement them for applications needing better photostability, sensitivity and wide variability. Magnetic nanoparticles will also broaden the area of already employed contrast agents and find more and more biomedical applications for their unique properties. The advantages of these types of nanoparticles are their low cost synthesis, huge modification possibilities and easy application. Such symbiosis of deep-rooted and modern methods will lead to improvement in early diagnostics as well as personalized therapy. However several difficulties have to be overcome before nanoparticles will be used as a routine tool, including the toxicity of metal and semiconductor nanoparticles and/or tissue autofluorescence in the visible spectral range. Nevertheless, the potential of the *in vivo* imaging is great and opens new possibilities for faster and better diagnostics on the one hand, and improved treatment strategies on the other hand.

## Figures and Tables

**Table 1.** The most important confocal microscopy techniques.

**Technique**	**Application**	**Nanoparticle (s)**	**Ref.**
Confocal Laser Scanning Microscopy	Cellular uptake/transport mechanisms study on Madin-Darby Canine Kidney (MDCK) cells	Coumarin-6 labeled nanoparticles	[100]
Confocal fluorescence microscopy	Cellular uptake/transport mechanisms study on red blood cells	Zwitterionic quantum dots	[101]
Confocal reflection microscopy	Improving of image contrast of tissue structures	60 nm gold nanospheres	[102]
Confocal Raman Microscopy	Cellular uptake and localization study on human hepatocarcinoma HepG2 cells	poly(lactide-co-glycolide) (PLGA) nanoparticles (NPs) and carbon nanotubes (CNTs)	[103]
Confocal interference scattering microscopy	Detection the orientation of individual silver nanorods and measure their relative distances	Silver nanorods	[104]

**Table 2.** The basic application of electron microscopy methods in NPs detection/visualization including the most common scanning (SEM) and transmission electron microscopy (TEM).

**Technique**	**Application**	**Nanoparticle (s)**	**Ref.**
Transmission electron microscopy (TEM)	Phase characterization of NPs	TiO_2_	[140]
Scanning electron microscopy (SEM)	General characterization	Silver nanoparticles	[117]
Scanning transmission electron microscopy (STEM)	General characterization	Platinum/ruthenium nanoparticles	[141]
Reflection electron microscopy (REM)	Nucleation control	Germanium nanoislands on ^111^Si surface	[142]
Low-voltage electron microscopy (LVEM)	General characterization and investigation of contrast mechanisms	Single-walled carbon nanotubes	[143]
